# New Draft Genome Sequence of the Ergot Disease Fungus Claviceps paspali

**DOI:** 10.1128/MRA.00498-20

**Published:** 2020-07-16

**Authors:** H. Oberti, E. Abreo, R. Reyno, M. Feijoo, S. Murchio, M. Dalla-Rizza

**Affiliations:** a Instituto Nacional de Investigación Agropecuaria (INIA), Unidad de Biotecnología, Estación Experimental INIA Las Brujas, Canelones, Uruguay; b Instituto Nacional de Investigación Agropecuaria (INIA), Laboratorio de Bioproducción, Estación Experimental INIA Las Brujas, Canelones, Uruguay; c Instituto Nacional de Investigación Agropecuaria (INIA), Programa Pasturas y Forrajes, Estación Experimental INIA Tacuarembó, Tacuarembó, Uruguay; d Centro Universitario Regional del Este (CURE), Polo de Desarrollo Universitario: Patogenicidad, Toxicidad y Genética en los Ecosistemas Pastoriles de la Región Este de Uruguay, Treinta y Tres, Uruguay; Vanderbilt University

## Abstract

Here, we report a new draft genome sequence of an isolate of the ascomycete Claviceps paspali that is responsible for ergot disease in grasses of the *Paspalum* genus. This new draft genome sequence will provide useful data for evaluating intraspecies and interspecies genome variation in *C. paspali* and other *Claviceps* genus members.

## ANNOUNCEMENT

Claviceps paspali (Sordariomycetes, Ascomycota) is a phytopathogenic causal agent of ergot disease in *Paspalum* spp. This disease is important for dairy and beef production because it affects highly productive, drought-resistant forage grasses, such as Paspalum dilatatum (1, 2). Ergot results in seed losses by seed replacement with the sclerotia of the fungus and also the production of tremorgenic toxins by the fungus, which are toxic to feeding animals (3, 4).

*C. paspali* ILB432 was isolated from sclerotia obtained from Paspalum urvillei-infected inflorescences collected near Portezuelo, Maldonado, Uruguay (global positioning system coordinates 34.888591S, 55.030316W), using a previously reported *C. paspali* isolation procedure (5). ILB432 was cultured at 26°C on *Claviceps* medium containing 36 g/liter potato dextrose agar, 2 g/liter yeast extract, 10 g/liter malt extract, 10 g/liter sucrose, and 5 g/liter agar (6). Phylogenetic analysis based on the partial gene coding for the second largest subunit of RNA polymerase subunit II (*RPB2*) showed that ILB432, like reference isolate RRC-1481, belongs to the most frequent lineage of *C. paspali* (5).

DNA was isolated from vegetative mycelia using the Quick-DNA fungal/bacterial kit (Zymo Research) following the manufacturer’s instructions and was sent to Macrogen, Inc. (Seoul, South Korea), for sequencing. DNA libraries of 500-bp inserts were generated with the TruSeq Nano DNA kit (Illumina), and 150-bp paired ends (PEs) were sequenced with the Illumina HiSeq 2500 platform.

Raw reads were trimmed to remove the adapter and low-quality sequences with Trimmomatic v0.36 (7). A total of 63,447,991 PE filtered reads were used for the *de novo* genome assembly using SPAdes v3.13.1 (8) with multiple k-mer sizes (21, 33, 45, 57, 69, 81, 93, 105, and 117) and the “–careful” option. The resulting assembly was analyzed using QUAST v4.6.1 (9). Species-specific repeats were inferred using the program RepeatModeler v2.0.1 (10), and Repeatmasker v4.1.0 (11) was employed to mask resulting repeats. Genome completeness was assessed using BUSCO v4.0.6 (12) (genome mode) against the Ascomycota_odb10 database. The newly assembled genome sequence contained 1,678 (98.4%) complete (single-copy and duplicated) BUSCO orthologs of the 1,706 present in the Ascomycota_odb10 database. Genome size and completeness results were similar to those of reference strain RRC-1481, which contains 1,653 complete BUSCO orthologs (Table 1).

**TABLE 1:** Detailed results from assembly of the two genomes of *C. paspali* isolates

Characteristics by category	Data for *C. paspali* isolate^*a*^:	
	ILB432	RRC-1481^*b*^
Assembly		
Estimated genome size (Mb)	28.9	28.9
Genome coverage (×)	653	54.4
GC content (%)	47.8	48.0
No. of contigs	1,030	2,152
*N*_50_ of contigs (bp)	66,931	26,979
No. of scaffolds with >200 bp	352	NA
*N*_50_ of scaffolds (bp)	146,886	NA
Longest scaffold (bp)	616,281	NA
No. of Ns per 100 kb	23.0	NA
Repeat sequences (%)	22.2	17.5
GenBank accession no.	JABAJK000000000	GCA_000223175.2
Completeness		
BUSCO (%)	98.4 (S), 0.0 (D), 0.5 (F), 1.1 (M)	96.9 (S), 0.0 (D), 1.2 (F), 1.9 (M)

a S, complete single copy; D, complete duplicated; F, fragmented; M, missing; NA, data not available.

b Assembly stats based on reference 13. BUSCO results based on this work analysis.

This improvement in sequencing methodology is reflected in the lower number of contigs and higher *N*_50_ values of this new genome sequence compared to those of the previous reference genome (Table 1). This study provides highly useful data for evaluating genome variation within *C. paspali* (7) and the *Claviceps* genus.

### Data availability.

This whole-genome shotgun project has been deposited in DDBJ/ENA/GenBank under the BioProject number PRJNA625338. This whole-genome shotgun project has been deposited at DDBJ/ENA/GenBank under the accession number JABAJK000000000. The version described in this paper is version JABAJK010000000. Raw reads are available under SRA accession number SRR11565825. The *RPB2* sequence is available under accession number MT348393. The pure isolate of *C. paspali* ILB432 is stored at the INIA Las Brujas fungal collection (ILB); for samples of the isolate, contact Eduardo Abreo at eabreo@inia.org.uy.
